# Overexpression of a Fungal β-Mannanase from *Bispora* sp. MEY-1 in Maize Seeds and Enzyme Characterization

**DOI:** 10.1371/journal.pone.0056146

**Published:** 2013-02-11

**Authors:** Xiaolu Xu, Yuhong Zhang, Qingchang Meng, Kun Meng, Wei Zhang, Xiaojin Zhou, Huiying Luo, Rumei Chen, Peilong Yang, Bin Yao

**Affiliations:** 1 Key Laboratory for Feed Biotechnology of the Ministry of Agriculture, Feed Research Institute, Chinese Academy of Agricultural Sciences, Beijing, People's Republic of China; 2 Biotechnology Research Institute, Chinese Academy of Agricultural Sciences, Beijing, People's Republic of China; 3 Institute of Food Crops, Jiangsu Academy of Agricultural Sciences, Nanjing, People's Republic of China; Wuhan Bioengineering Institute, China

## Abstract

**Background:**

Mannans and heteromannans are widespread in plants cell walls and are well-known as anti-nutritional factors in animal feed. To remove these factors, it is common practice to incorporate endo-β-mannanase into feed for efficient nutrition absorption. The objective of this study was to overexpress a β-mannanase gene directly in maize, the main ingredient of animal feed, to simplify the process of feed production.

**Methodology/Principal Findings:**

The *man5A* gene encoding an excellent β-mannanase from acidophilic *Bispora* sp. MEY-1 was selected for heterologous overexpression. Expression of the modified gene (*man5As*) was driven by the embryo-specific promoter ZM-leg1A, and the transgene was transferred to three generations by backcrossing with commercial inbred Zheng58. Its exogenous integration into the maize embryonic genome and tissue specific expression in seeds were confirmed by PCR and Southern blot and Western blot analysis, respectively. Transgenic plants at BC3 generation showed agronomic traits statistically similar to Zheng58 except for less plant height (154.0 cm vs 158.3 cm). The expression level of MAN5AS reached up to 26,860 units per kilogram of maize seeds. Compared with its counterpart produced in *Pichia pastoris*, seed-derived MAN5AS had higher temperature optimum (90°C), and remained more β-mannanase activities after pelleting at 80°C, 100°C or 120°C.

**Conclusion/Significance:**

This study shows the genetically stable overexpression of a fungal β-mannanase in maize and offers an effective and economic approach for transgene containment in maize for direct utilization without any purification or supplementation procedures.

## Introduction

Mannan is the second most abundant hemicellulosic polysaccharide after xylan in nature [1]. It consists of a backbone of β-1,4-linked mannose or a combination of glucose and mannose residues, and the mannan residues are often substituted with α-1,6-galactose as side chains and acetylated at the *O*-2 and *O*-3 positions depending upon their origin [2], [3]. The diversity of the mannan structure allows their wide range of physico-chemical properties [4] and classification into four families: mannan, glucomannan, galactomannan and galactoglucomanan [5]. Pure mannans are insoluble, when the mannose residues are replaced by glucoses in glucomannans or by galactoses in galactomannans, their water-solubility is increased [6]. In animal feed, mannans have been defined as one of the intense anti-nutritional factors [7]; they often combine with water, increase the viscosity of chyme, block the intestinal surface partially, and thus reduce the feed conversion and limit the efficiency of carbohydrate utilization [8], [9]. Moreover, these anti-nutritional factors can cause flatulence, and sometimes are responsible for digestive disorders and metabolic diseases [10], [11]. To overcome these problems, β-mannanases are generally supplemented into animal diets to digest anti-nutritional factors, stimulate digestion, and minimize the negative effects of specific components of feed ingredients on nutrient digestion [10], [12]–[15]. On the other hand, the hydrolysis products from mannans―mannose oligosaccharides have been reported to possess immunogenic potential that strengthens the immunity of animals from diseases [16].

There are three enzymes involved in the complete decomposition and conversion of mannan; of them, β-mannanase (endo-1,4-β-mannanase; EC 3.2.1.78) is the crucial enzyme that catalyzes the random hydrolysis of β-d-1,4-mannopyranosyl linkages within the backbone [1], [17], and exo-β-mannanase and β-mannosidase are auxilary [14]. Based on the amino acid sequence and structural similarity among catalytic domains (http://www.cazy.org/), majority of β-mannanases are grouped into glycoside hydrolase (GH) families 5, 26, and 113 [18]. At present, feed enzymes are mostly sourced from microorganisms [19], [20], and they are generally produced in prokaryotic (eg. *Escherichia coli* and *Bacillus subtilis*) or eukaryotic (*Pichia pastoris*, *Trichoderma* spp., *Aspergillus* spp., etc) expression systems for commercial purpose [13], [21], [22]. So far several plant expression systems have been developed to produce enzymes. For example, Ziegler *et al.* [23] and Jiang *et al.* [24] produced an endoglucanase from *Acidothermus cellulolyticus* and a cellulase from *Thermobifida fusca* in the leaves of *Arabidopsis thaliana* and *Nicotiana tabacum*, respectively. A radish defensin has been expressed in transgenic wheat (*Triticum aestivum* L.), leading to the increased resistance to *Fusarium graminearum* [25]. Exogenous β-mannanase genes have been proved to be successfully expressed in higher plants. Hoshikawa *et al.* [26] expressed an endo-β-mannanase gene from deep-sea *Bacillus* sp. JAMB-602 in tobacco that showed enhanced resistance against *Fuscarium oxysporum*. Agrawal *et al.* [27] also expressed a β-mannanase gene of *Trichoderma reesei* in tobacco chloroplasts for wood biomass hydrolysis.

Maize as the main ingredient of animal feed is an ideal natural bio-reactor in which a phytase gene from *Aspergillus niger* 963 has been successfully expressed with the phytase activity of 2,200 U/kg in seeds [28]. The aim of this study was to develop a genetically stable maize line that has high β-mannanase activity and excellent properties. The mannanase gene, *man5A*, from acidophilic *Bispora* sp. MEY-1 [29] was selected due to the excellent properties of its coding protein, such as high activity and stability over the physiological pH (1.0–6.0) of animal digestive tract, high temperature optimum (65°C), good stability at 60°C, and strong resistance towards proteases. Maize is a renewable resource; the development of transgenic maize will not only reduce the loss of resources and simplify the production process, but also provide an environmentally friendly approach to produce feed enzymes.

## Materials and Methods

### Plant materials

The widely used and highly productive maize variety Hi-II [30], [31] was used for genetic transformation and mannanase production. The isolated immature embryos were preserved on N6 1-100-25 medium [32] for callus induction. Maize Hi-II callus has excellent regeneration capacity and can respond reasonably well under a wide variety of *in vitro* culture conditions. The commercial maize inbred-line Zheng58 was genetically stable and was used to produce progenies.

### Codon modification of the β-mannanase gene

The DNA sequence of native *man5A* from *Bispora* sp. MEY-1 (EU919724) contained an N-terminal Ser/Thr-rich sequence and a putative signal peptide-coding sequence [29]. After removal of these sequences, codon optimization was performed according to the translationally optimal codon usage of maize [33], [34]. Codon adaptation index (CAI) and GC content analysis were used to evaluate the gene coding sequence and codon usage for the prediction of gene expression level. The optimized gene was synthesized by Genscript (Nanjing, China) and was cloned into pUC57MCS. Because *man5A-sst* contained restriction sites of *Bam*HI, *Sma*I and *Xma*I that are unsuitable for direct cloning into expressing vector, these sites were removed by two pairs of primers, 1417-200mutF/1417-200mutR and 1417-800mutF/1417-800mutR (see Table 1). The newly modified gene was named *man5As* that encoded the same amino acid sequence as the N-terminus truncated *man5A-sst* did [29].

**Table 1:** Primers used in this study.

Primer name	Primer sequence (5′→3′)	Size (bp)
20754-186F	AACTCCGGCTTCGCGGACTC	20
20754-398R	TTCCTGGCAAATCACTCGGTGTATC	25
1417man-F^a^	CTAGGATCCCCGGCGTACCCCATCTGC	27
1417man-R^a^	TAACCCGGGCTACACCGGCTTCTCCAGCATG	31
1417-200mut-F	AGGATCTGGGGGTTCGGCAGCGTCAACACGGACCCCGGCCCCGGCACGGTCTTC	54
1417-200mutR	GACGCTGCCGAACCCCCAGATCCTGACCACCTGGAGCTGGGTGTT	45
1417-800 mutF	TCGACGTGGATTCTGCAGCACAACGAGGTG	30
1417-800mutR	CACCTCGTTGTGCTGCAGAATCCACGTCGAGCCCCAG	37
AC326F	ATGTTTCCTGGGATTGCCGAT	21
AC326R	GCATCACAAGCCAGTTTAACC	21

a Restriction sites incorporated into primers are underlined.

### Plasmid construction

**Figure 1:**
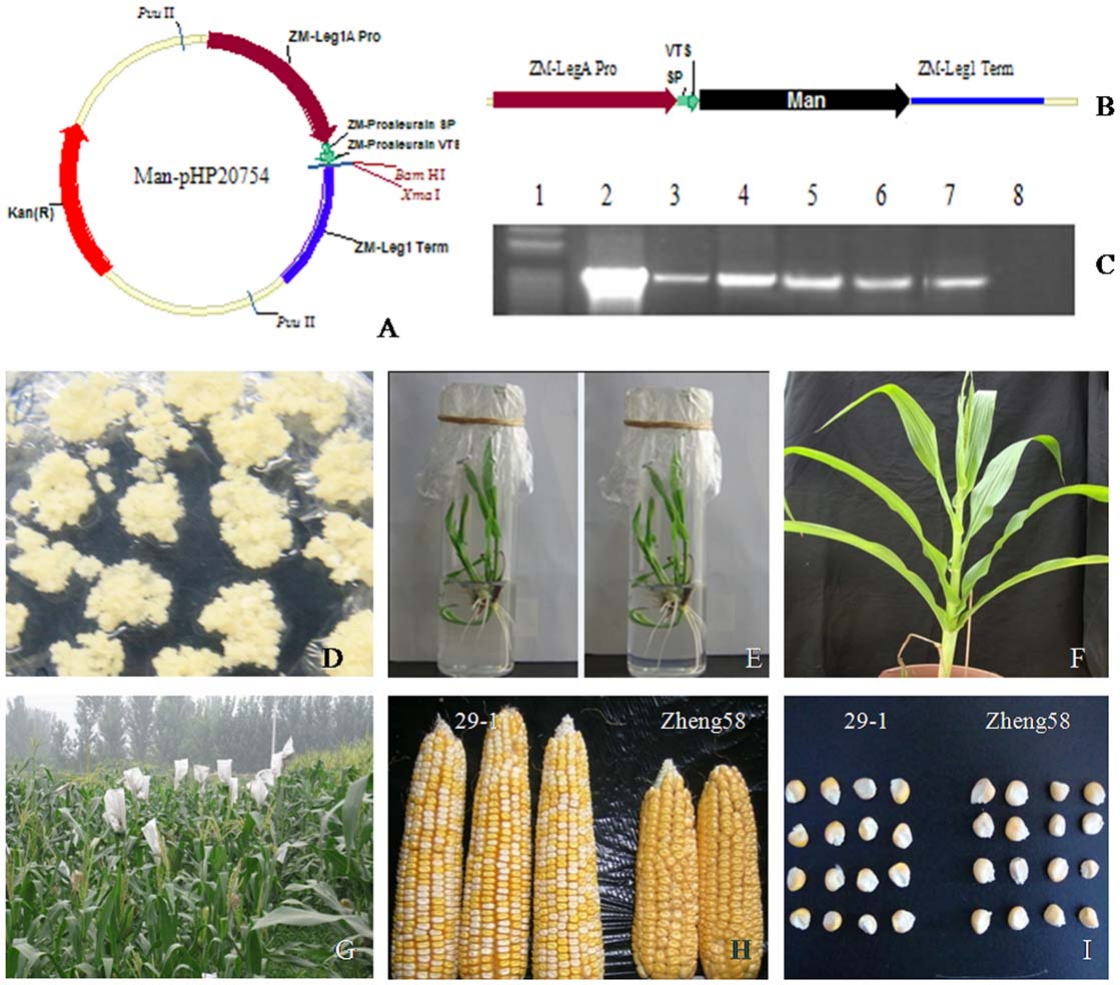
Construction of the recombinant vector and regeneration of transgenic maize. **A**) The recombinant expression vector pHP20754-*man5As*. **B**) The chimeric gene cassettes for expression in maize. **C**) PCR analysis of five putative calli. Lane 1, the DNA molecular weight markers; lane 2, the expression vector pHP20754-*man5As* (positive control); lane 3–7, the calli of transgenic maize Hi-II; lane 8, the calli of non-transgenic maize Hi-II (negative control). **D**) Embryogenic calli in selective medium. **E**) Plantlets in rooting medium. **F**) Regenerated maize plants in the greenhouse. **G**) Transgenic maize in fields. **H**) Ears of generation T1 of transgenic plant and non-transgenic maize Zheng58. **I**) Seeds of generation T1 of transgenic plant and non-transgenic maize Zheng58.

The vector pHP20754 consists of the ZM-leg1A promoter, the ZM-leg1 terminator, the maize proaleurain signal peptide (SP) and the vaculoe targeting sequence (VTS) (Figure 1A). The ZM-leg1A promoter is endosperm specific. A pair of specific primer (1417man-F and 1417man-R contaning the *Bam*HI and *Xma*I sites, respectively; Table 1) was used to amplify the mutant gene *man5As* from pUC57MCS. The PCR conditions were as follows: 5 min at 95°C, followed by 30 cycles of 95°C for 30 s, 55°C for 30 s, and 72°C for 90 s. The PCR products were purified with a DNA purfication kit (TaKaRa, Osaka, Japan) and were ligated to the vector pEASY-T_3_ (TransGen, Beijing, China) for sequencing. Both the vector pHP20754 and *man5As* were digested with *Bam*HI and *Xma*I, and ligated together with T4 DNA ligase to construct the chimeric gene cassettes for expression (Figure 1A). The recombinant vector pHP20754-*man5As* was then digested with *Pvu*II for transformation. All the restriction endonucleases and T4 DNA ligase were purchased from New England Biolabs (Ipswich, MA).

The plasmid pHP17042BAR carrying the maize histone H2B promoter, the maize Ubiquitin 5′-UTR intron-1, the *bar* gene from *Streptomyces hygroscopicus* and the potato protease II terminator [28] was used as the selectable marker for transformation. The *bar* gene was excised from pHP17042BAR by *Hind*III, *Xho*I and *Sac*I for screening of positive transgenic plants.

### Transformation, selection and regeneration

The concentrations of *man5As* and the *bar* gene were adjusted to 200 ng/µl. The recombinant vector was then transformed into maize Hi-II cells with high-velocity microprojectiles (Bio-Rad, Hercules, CA) wrapped by DNA molecules [35], [36]. After recovery, embryonic calli were transferred onto the selective medium supplemented with bialaphos as a selectable marker. The positively transformed calli were cultivated in differentiation medium and rooting medium in succession. Seedlings (T0 plants) were transplanted into greenhouse. Zheng58 with stable inheritance was used as the male parent to produce T1 seeds. Backcross method was used to produce BC1 to BC3 generations in field.

### Analysis of plant agronomic trait and seed composition of BC3 generation

Ten of each transgenic (BC3 generation) and non-transgenic (Zheng58) maize plants were randomly selected for agronomic trait analysis. As shown in Table 2, data of nine traits of each individual plant were recorded. T test was used to compare the difference of transgenic and non-transgenic data. Contents of moisture, crude protein, fat, fiber, ash, nitrogen free extract and each amino acid of the maize seeds of BC3 generation and Zheng58 were analyzed according to the standard protocol.

**Table 2:** Agronomic traits of transgenic and non-transgenic maizes in BC4 generation.

Agronomic traits	Transgenic plant	Zheng58	*p* value
Primary ear height (cm)	55.4±5.7	54.5±4.7	>0.05
Plant height (cm)	154.0±7.2	158.3±4.4	0.028
Ear length (cm)	17.9±0.3	18.1±0.2	>0.05
Ear diameter (cm)	5.4±0.1	5.4±0.1	>0.05
Leaves above primary ear	4.5±0.5	4.6±0.5	>0.05
Tassel branch	6.2±1.3	5.3±1.7	>0.05
Rows/ears	12.6±1.0	13.0±1.1	>0.05
Kernels/ear	30.5±1.6	30.4±1.1	>0.05
100 kernel weight (g)	38.5±0.3	38.5±0.3	>0.05

### PCR detection of exogenous gene integration

Genomic DNA was extracted from the maize leaves of generations T1 to BC3 using the CTAB method [37]. The specific primers 1417-800mutF and 20754-398R (Table 1) were used to confirm the positive lines. The recombinant plasmid pHP20754-*man5As* and the genomic DNA of Zheng58 were used as the positive and negative controls, respectively. The PCR conditions were: initial denaturation at 94°C for 5 min, followed by 32 cycles of 30 s at 94°C, 30 s at 57°C and 45 s at 72°C. Primers AC326F and AC326R (Table 1) specific for the *actin* gene were used to check the quality of genomic DNA. The PCR products were analyzed on a 1.2% (w/v) agarose gel. All maize leaves of generation T1 to BC3 were tested.

### Southern blot

Five grams of maize leaves of T1 to BC3 generations of transgenic events 22 and 29 were ground with liquid nitrogen, and genomic DNA was extracted with the CTAB method. Genomic DNA of Zheng58 was used as the negative control. About 50 µg of genomic DNA was digested by *Hind* III and *Bam*HI and was separated on a 0.8% (w/v) agarose gel. The agarose gel was transferred onto a hybond-N^+^ nylon membrane (GE Healthcare, Uppsala, Sweden) with a Trans-Blot SD system by UV-crosslinking. A digoxin-labled probe containing a 770 bp fragment of *man5As* was used for in-situ hybridization. Immunologic process followed the instructions of DIG-High Prime DNA Labeling and Detection Starter Kit II (Roche, Indianapolis, IN).

### Western blot

Five milligrams of lyophilized purified MAN5A-SST produced in *P. pastoris* GS115 [29], [38] was used for the production of polyclonal antibody in rabbits by Laboratory Animal Center, Institute of Genetics and Developmental Biology, Chinese Academy of Sciences (Beijing, China). Two hundred microliters of polyclone was diluted by 800 µl of 1× PBS, pH 7.4. After addition of 1 ml of protein extract from Zheng58 seeds, the mixture was incubated at 37°C for 2 h, followed by centrifugation at 9,167× *g* for 10 min. The supernatant was mixed with 1 ml of yeast cells transformed with the empty vector pPIC9. After centrifugation at 9,167× *g* for 10 min, the supernatant was dialyzed successively against 1× PBS (pH 7.4) and 0.025 M acetic acid (pH 4.0), and was collected by centrifugation.

After drying in the sun or in an oven, maize seeds of the transgenic lines and Zheng58 were smashed into powder with a high-throughput tissue homogenizer (2010 Geno/Grinder, SEPX CertiPrep, Metuchen, NJ). Seed powder (30 mg of each sample) was put into a 1.5 ml tube containing 300 µl of 100 mM KCl, pH 1.5 (extraction buffer), and agitated on a shaker for 1 h (20°C, 350 rpm). Supernatant of seed extract (150 µl) of each sample was incubated with pro-cooled acetone at the ratio of 1∶2 for 30 min followed by centrifugation at 14,000× *g* for 15 min. After removing the supernatant, 30 µl of ddH_2_O was added to dissolve the seed protein. The protein sample was divided into two equal parts. One part was deglycosylated with endo-β-*N*-acetylglucosaminidase (Endo H) according to the supplier's instructions (New England Biolabs), the other remained intact. Protein extract of Zheng58 and purified MAN5A-SST from *P. pastoris* were used as the negative and positive controls, respectively. Proteins from the stem, root and leaf of a transgenic plant of generation BC1 were extracted and used for tissue specificity analysis.

Proteins were separated on SDS–PAGE (12% acrylamide) and transferred onto PVDF membrane (Pall, Port Washington, NY). The pretreated first antibody was added into the membrane confining liquid (TIANGEN, Beijing, China) for prehybridization. The goat anti-rabbit IgG labled with alkaline phosphatase (Abcam, Hong Kong, China) was used as the secondary antibody. BCIP/NBT kit (Zomanbio, Beijing, China) was used for color development. To identify the proteins, bands were excised from the gel and analyzed using matrix assisted laser desorption/ionization time of flight mass spectrometry (MALDI-TOF) at Tianjin Biochip Corporation (Tianjin, China).

### Evaluation of the β-mannanase activity

Crude proteins of five randomly selected seeds were extracted with extraction buffer as described above, and the supernatant was subjected to β-mannanase activity assay [17], [29]. One unit of β-mannanase activity was defined as the amount of enzyme to release 1 µmol reducing sugar per minute at the assay conditions (pH 1.5, 65°C, 10 min). β-Mannanase activities of generations T1, BC1 and BC2 of transgenic maize and Zheng58 were all evaluated in triplicate.

### Property comparison of MAN5AS and MAN5A-SST

Enzyme characterization of the crude proteins extracted from BC2 seeds and *P. pastoris* was carried out as Luo *et al.* described for MAN5A-SST [29]. The pH optimum for β-mannanase activity was determined at 65°C for 10 min in the reaction buffers with pH ranging from 1.0 to 10.0. The optimal temperature was examined at 30–95°C in 100 mM KCl-HCl (pH 1.5). pH stability was determined by measuring the residual activity under standard conditions (pH 1.5, 65°C, 10 min) after preincubating the enzyme at pH 1.0–11.0 at 37°C for 1 h. Thermostability was measured under the standard conditions as mentioned above after being incubated at 60°C or 90°C for various periods without substrate, respectively.

### Evaluation of anti-inactivation stability over feed pelleting process

Feed pelleting was carried out with a twin-screw extruder (DSE-25 Extruder Lab-Station Brabender OHG, Duisburg, Germany). Part of the maize seeds of each generation from T1 to BC3 were mixed and extruded at 80°C, 100°C or 120°C, respectively. β-Mannanase activities and dry matter content (DM) values were determined before and after pelleting. Zheng58 seeds were treated as the non-transgenic control. Equal amounts of crude MAN5A-SST based on β-mannanase activities were added into Zheng58 seeds, followed by pelleting treatment as described above. And the loss rates of mannanase activities were both detected after pelleting.

## Results

### Construction and transformation of embryo-specific vector harboring *man5As* gene

The CAI value and GC content of *man5A-sst* were 0.72 and 51.41%, respectively. After codon optimization and gene modification, the CAI value and GC content of *man5As* was increased to 0.94 and 64.99%, respectively. These higher values are better for exogenous gene expression in maize. As a result, both native *man5A-sst* and synthetic *man5As* were 1095 bp in length, shared 85.9% nucleotide sequence identity and encoded similar 365 amino acid residues with the expected protein weight of 40.5 kDa.

*man5As* was inserted into the expression vector pHP20754 between the embryo-specific ZM-leg1A promoter and ZM-leg1 terminator (Figure 1B), which is a transcriptionally active spacer region that allows highly efficient transgene expression. To identify the positive transformants, gene fragments of 450 bp (Figure 1C) were amplified from the calli of maize Hi-II regenerated on bialaphos medium (Figure 1D), using the specific primers of *man5As* (see Table 1).

### Plant regeneration and phenotypic evaluation

The regenerated young plants described above showed good growth in the rooting medium (Figure 1E) and in the greenhouse (Figure 1F). Plants of two independent transgenic events 22 and 29 were cultivated in field from generation BC1 (Figure 1G). A total of 21 independent transgenic lines were obtained. The transformation efficiency was 35%, and 3,548 T1 seeds were harvested, 292 of which were backcrossed with commercial Zheng58 to produce their progenies. As shown in Figure 1H and 1I, T1 ears and seeds of a transgenic plant showed significant phenotype difference from Zheng58. That may be due to the heterotic vigor of T1 plants. And the heterotic vigor would subside generally in the later generations because of the successive backcross with Zheng58. Agronomic traits of transgenic plants of generation BC3 were compared with that of non-transgenic Zheng58. Of nine traits analyzed (Table 2), only one trait-plant height showed significant difference (154.0 cm vs. 158.3 cm, *p*<0.05). The result suggests that maize with transgene had almost the same phenotype as wild type plant. Comparison of the composition of generation BC3 and Zheng58 seeds showed that there is no significant difference between transgenic and non-transgenic maize seeds (Table S1).

### Determination of exogenous gene integration

**Figure 2:**
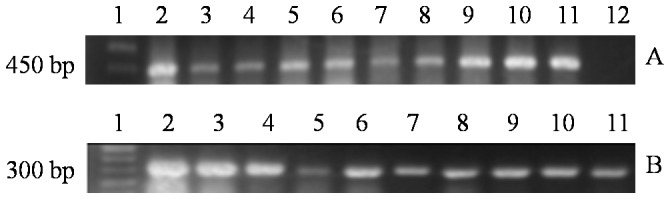
PCR analysis of the genomic DNA from leaves of generation BC1 of transgenic and non-transgenic plants. **A**) PCR detection of the gene *man5As*. Lane 1, the DNA molecular weight markers; lane 2–10, the transgenic plants; lane 11, the vector pHP20754-*man5As*; lane 12, the non-transgenic Zheng58. **B**) PCR detection of the gene *actin*. Lane 1, the DNA molecular weight markers; lane 2–10, the transgenic plants; lane 11, the non-transgenic Zheng58.

PCR assay with primers specific for *man5As* was used to evaluate the inheritance of transgenic maizes from generation T1 to BC3. PCR results of *actin* gene (∼300 bp) indicated the high quality of genomic DNA (Figure 2B). Gene fragments of about 450 bp were detected in the transformation events 29 and 22 (Figure 2A). The positive rates of all generations based on PCR results (Table 3) showed a rising trend, suggesting the stability for future generations.

**Table 3:** Positive rates of four generations of two transgenic events based on PCR analysis.

Event	Generation	No. of positive plants	No. of plants	Positive rate (%)
	T1	17	52	33
	BC1	295	647	46
	BC2	276	587	47
	BC3	11	25	44
22	T1	7	49	14
	BC1	57	164	35
	BC2	33	112	29
	BC3	3	9	33

**Figure 3:**
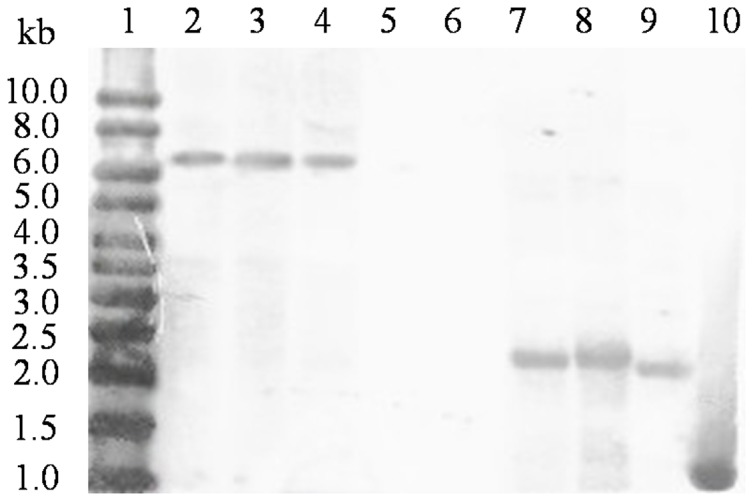
Southern blot analysis of MAN5AS in three transgenic plants of event 22 after digestion with *Hind*III and *Bam*HI. Lane 1, the DIG-labeled molecular weight markers; lane 2–4, MAN5AS with *Bam*HI digestion; lane 5 and 6, non-transgenic Zheng58 digested by *Bam*HI and *Hind*III, respectively; lane 7–9, MAN5AS with *Hind*III digestion; lane 10, the digested expression cassettes as a positive control.

To confirm the gene integration and the copy number of *man5As* in transgenic plants, the genomic DNAs of three positive transgenic plants of event 22 were analyzed by Southern blot after restriction digest with *Hind*III and *Bam*HI. A band of ∼1.4 kb was detected in the positive lane, but not in non-transgenic Zheng58. *Hind*III cut the chimeric *man5As* twice that relieved an internal fragment of 2.4 kb from the gene expression cassettes (Figure 3). *Bam*HI and *Xma*I were the ligation sites of transgene *man5As* and vector pHP20754. After *Bam*HI digest, only one band migrated (Figure 3), indicating that there is only one copy of *man5As* in event 22.

### Evaluation of site-specific expression

**Figure 4:**
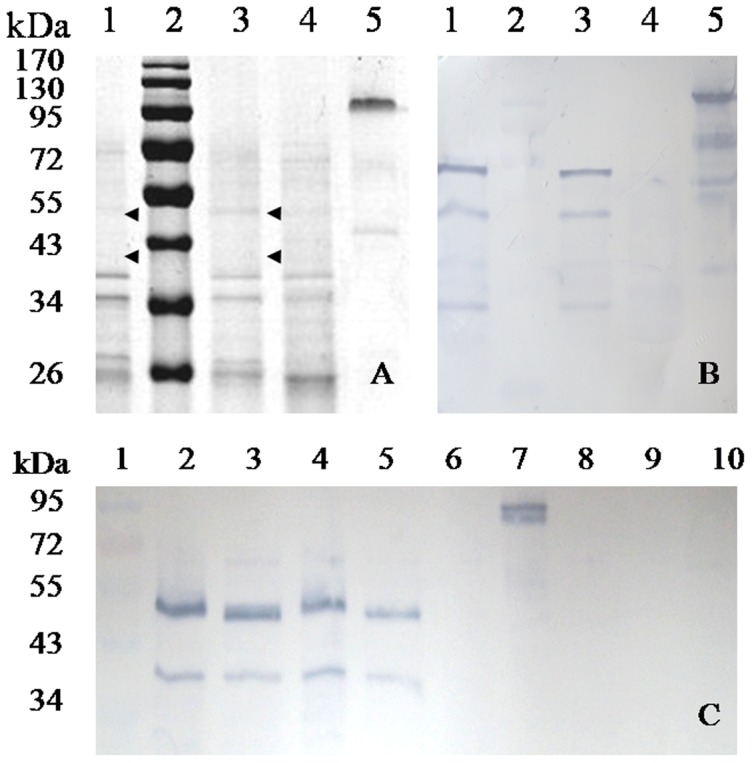
Analysis of recombinant MAN5AS from two transgenic maizes. **A**) SDS–PAGE. **B**) Western blot. Lane 1 and 3, the transgenic maize; lane 2, the protein molecular markers; lane 4, the non-transgenic Zheng58; lane 5, the purified MAN5A-SST produced in *P. pastoris*. **C**) Specific promoter analysis. Lane 1, the protein molecular markers; lane 2 and 4, the protein isolated from seeds of T042-5 and T041-20; lane 3 and 5, the two transgenic plant proteins pretreated with Endo H; lane 6, the protein isolated from seeds of non-transgenic Zheng58; lane 7, the purified MAN5A-SST produced in *P. pastoris*; lane 8–10, the proteins collected from root, stem and leaf tissue of a transgenic plant.

To determine the expression efficiency of exogenous MAN5AS, proteins were extracted from two BC2 plants (T042-5 and T041-20) of event 29 that had high β-mannanase activities (33,468 U/kg, 32,592 U/kg). Compared with the image on SDS–PAGE (Figure 4A), three main bands were identified on the PVDF membrane after hybridization with the untreated primary antibody (Figure 4B). Only two bands of approximately 40 kDa and 50 kDa were developed when the primary antibody was pre-hybridizated with the proteins extracted from *P. pastoris* harboring the empty vector or Zheng58 (Figure 3C). Both bands were verified to be MAN5AS through MALDI-TOF analysis (the protein scores C.I. % are 99.84614% and 92.39621% for MAN5AS and MAN5A-SST, respectively). With Endo H treatment, the ∼50 kDa band showed some reduction in molecular weight while the ∼40 kDa that was similar to the predicted molecular weight kept intact. No band was detected on negative control. The positive control, MAN5A-SST expressed by *P. pastoris*, showed a band of about 90 kDa, the same as that reported in [29]. Proteins extracted from the root, stem and leaf of the positive lines had no objective band (Figure 4C), indicating the tissue specificity of MAN5AS by using the endosperm specific ZM-leg1A promoter. This promoter made exogenous MAN5AS specifically expressed in the seeds of transgenic maize and could lessen the potential impairment to the plants. Moreover, MAN5AS present in seeds are more convenient for storage and transportation.

### Evaluation of seed-derived β-mannanase activity

Positive transgenic plants of transgenic event 22 and 29 as confirmed by PCR were selected for β-mannanase activity assay. Seed β-mannanase activities of T1 to BC3 plants were assessed using the DNS method (Table 4). Compared with the non-transgenic Zheng58 that had an average β-mannanase activity of 1,265 U/kg of seeds, T1 seeds of two events showed approximately 20-fold activities of Zheng58. Both events showed significant declined β-mannanase activities in BC1 seeds but recovered in generation BC2. The average β-mannanase activities of BC2 and BC3 seeds were about 10,000 U/kg, and the rate of seeds with β-mannanase activity over 5,000 U/kg were about 42%. The result further confirmed that *man5As* transgene is genetically stable over generations.

**Table 4:** β-Mannanase activities of four generations of two transgenic events.

Event	Generation	No. of seeds with β-mannanase activity (U/kg)						Maximal activity (U/kg)	Average activity (U/kg)
		>50,000	10,000–50,000	5,000–10,000	1,000–5,000	<1000	Total		
29	T1	1	5	0	1	2	9	47,620	26,860
	BC1	0	87	40	28	85	240	25,530	7,110
	BC2	0	97	21	111	80	310	41,916	11,781
	BC3	0	11	7	11	11	40	17,788	5,810
22	T1	4	3	1	8	4	29	85,365	20,017
	BC1	0	3	0	1	16	20	18,412	2,008
	BC2	0	29	8	35	19	81	38,584	8,146
	BC3	0	14	1	1	4	20	44,511	15,295
Zheng58	/	0	0	0	6	4	10	2,870	1,265

The β-mannanase activities of BC1 ears of 15 transgenic lines of event 29 and 5 transgenic lines of event 22 were also tested (Table 5). The β-mannanase activities of BC1 ears varied a lot (from 435 to 28,537 U/kg), even within the same transgenic line. Because BC1 seeds were backcrossed with non-transgenic Zheng58, the activity variation in ears of the same transgenic line may be due to segregation. The average activity of all tested ears was 9,377 U/kg. T44-7-31 and T44-20-38 showed the highest expression level of *man5As* in transgenic event 29 (12,827 and 15,235 U/kg on average, respectively), and T62-18-54 of event 22 had the highest β-mannanase activity (18,974 U/kg) of all transgenic lines tested.

**Table 5:** β-Mannanase activities of BC1 seeds of 20 transgenic lines.

Transgenic line	β-Mannanase activity (U/kg)			Transgenic line	β-Mannanase activity (U/kg)		
	Ear 1	Ear 2	Ear 3		Ear 1	Ear 2	Ear 3
Event 29				T45-17-43	12,614	NA	NA
T44- 7-31	24,780	12,500	1,200	T46- 3-44	11,144	4,611	1,553
T44- 8-32	16,170	10,780	2,080	T46- 8-45	7,433	1,940	741
T44- 9-33	11,790	5,440	1,170	T46- 9-46	13,506	13,148	915
T44-10-34	10,790	1,940	1,510	T46-10-47	11,709	9,361	NA
T44-19-37	14,652	10,619	5,819	Event 22			
T44-20-38	28,537	11,416	5,753	T58- 2-48	19,420	1,444	NA
T45- 2-39	10,319	2,374	NA	T58- 5-49	1,825	1,033	NA
T45- 6-40	24,472	5,826	2,270	T58- 6-50	13,973	1,342	NA
T45- 8-41	25,410	3,180	718	T59-15-51	12,429	3,740	NA
T45-14-42	16,414	7,076	435	T62-18-54	26,764	11,184	NA

NA, not assayed.

### Characterization of *P. pastoris*-derived MAN5A-SST and maize seed-derived MAN5AS

**Figure 5:**
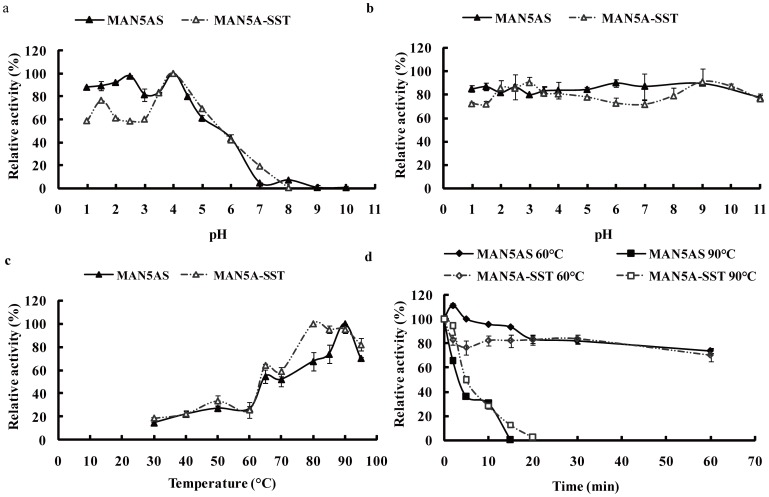
Property comparison of recombinant β-mananases expressed in maize (MAN5AS) and *P. pastoris* (MAN5A-SST). **a**) pH-dependent activity profiles of MAN5A-SST and MAN5AS at 65°C. **b**) pH stability of MAN5A-SST and MAN5AS activities at 37°C for 1 h. **c**) Temperature-dependent activity profiles of MAN5A-SST and MAN5AS at pH 1.5. **d**) Thermostability of MAN5A-SST (dashed line) and MAN5AS (solid line) at 60°C (diamond) or 90°C (square) at pH 1.5. Error bars represent the standard deviation of triplicate measurements.

The crude proteins of transgenic BC2 seeds and *P. pastoris* fermentation broth were characterized and compared (Figure 5). Both crude enzymes had pH optimum at 4.0, remained active at 1.0–6.0, and retained stable at pH 1.0–11.0. The temperature optimum of MAN5AS was 90°C, 10°C higher than that of MAN5A-SST. Thermostability of MAN5AS and MAN5A-SST were similar, retaining ∼80% activity at 60°C for 60 min and completely inactivated at 90°C for 20 min.

### Evaluation of anti-inactivation stability in feed pelleting

The β-mannanase activities of MAN5AS and MAN5A-SST were determined after feed pelleting at 80°C, 100°C or 120°C, respectively (Table 6). Both transgenic maize and Zheng58 had the DM of 89%. The initial β-mannanase activities in transgenic line or in Zheng 58 by supplementation of MAN5A-SST were set to 2,760 U/kg. After pelleting at each of the tested temperatures, MAN5A-SST lost more activities than MAN5AS, indicating that MAN5AS was more stable over pelleting process than MAN5A-SST.

**Table 6:** β-Mannanase activities of MAN5AS and MAN5A-SST before and after feed pelleting.

	β-Mannanase activity (U/kg)		Activity loss (%)
	Before pelleting	After pelleting	
MAN5AS			
80°C	2,760	1,490	46
100°C	2,760	1,370	50
120°C	2,760	1,240	55
MAN5A-SST			
80°C	2,760	690	75
100°C	2,760	1,130	59
120°C	2,760	1,150	58

## Discussion

With the agriculture and economic development, natural and conventional biosources are hard to satisfy the demands of our life. Transgenic plants are being developed for wide commercial and environmental values. Moreover, genetic engineering techniques have been used to improve the qualities of agriculture crops worldwide [39]. In 2011, the plantation area of transgenic plants reached about 160 million hectares and was distributed in 29 countries (http://www.isaaa.org/resources/publications/briefs/41/executivesummary/default.asp). In the feed industry, most of the genetically modified crops are planted for their phenotypic trait of insect resistance, disease resistance, herbicide resistance, etc. And they are mainly used as source of energy and proteins because of their low cost. However, genetically modified crops designed for increasing the nutritive ratio in animal feeds are scarce. Feed enzymes are generally produced by microbial fermentation. This process is flexible and convenient, but accompanies with a high cost in equipment and energy consumption. Moreover, feed enzymes are conventionally added into feed through a complex process of isolation, purification and supplementation, which require more energy and resources. Thus it's a good way to produce feed enzymes in feed grains directly, not involving extra industrial production. Maize as the major ingredient of animal feed (about 50%) represents a more important bioreactor to produce feed enzymes than other grains. To improve the phytate utilization in livestock, Chen *et al.* [28] had successfully overexpressed an *Aspergillus niger* phytase gene in maize seeds. This transgenic maize has been authorized to be the first phytase transgenic plant in China and set the basis for development of more transgenic plants for feed enzymes. In this study, we expressed a fungal β-mannanase from *Bispora* sp. MEY-1 in maize. To our knowledge, this is the first report of expression of fungal mannanase in forage crop and its direct utilization in animal feed.

Substitution of rare codons with preferred codons is able to enhance and stabilize expression of foreign genes in plants [40]. Using this method, Hiwasa-Tanase *et al.* has successfully achieved high-level expression of a miraculin gene in transgenic tomato [41]. Li *et al.* improved the Bt cry1Ah gene expression in transgenic maize through codon optimization [42]. In this study, we optimized *man5As* from *man5A* of *Bispora* sp. MEY-1 by using the same code usage method, and expressed the gene in maize by transformation into the immature embryos of maize Hi-II. In comparison with wild-type Zheng58 (Figure 1), transgenic lines of *man5As* showed normal phenotype and similar characteristics (Table 2) indicated that the inserted exogenous gene has no significant difference on most of the basic traits. Thus transformation of *man5As* in maize had no negative impact on the plant growth. Moreover, seed composition of transgenic and non-transgenic maize had little difference, which could be alleviated after five or six times of backcrossing with Zheng 58 and three or four times of selfcrossing. Further PCR and Southern blot analysis of the maize genomic DNA showed the genetic stability of one-copy *man5As* over times (Table 3, Figure 3). The results indicated that microprojectile bombardment is efficient and reliable to transform exogenous genes into the immature embryos of maize Hi-II.

In a previous study a, *Trichoderma reesei* β-mannanase gene was expressed in tobacco chloroplasts and the enzyme activity was 25,000 U/kg of fresh old leaves [27]. To achieve high-level expression of *man5As* in maize, several strategies have been utilized in combination, including (1) a synthetic gene with preferred maize codons; (2) a strong tissue-specific promoter; (3) an excellent transformation receptor with high competence and regeneration capacity that improves the transformation efficiency; (4) a positive effect by propagating from transgenic lines with high enzyme activities [43]. As a result, the average β-mannanase activities of maize seeds of four generations ranged from 2,008 to 26,860 U/kg (Table 4), several times over the non-transgenic Zheng58. This enzyme activity is high enough to substitute the microbial β-mannanase supplement in animal feed.

Two protein bands (∼40 and ∼50 kDa) were detected by Western blot analysis and were both identified to be MAN5AS through mass spectrometry. The result suggests that both bands are two posttranslational isozymes of MAN5AS in maize seeds. Post-translation modification is very common in eukaryotic proteins. Dirk *et al.* had reported multiple isozymes of an endo-β-mannanase in monocotyledonous plants [44]. *N*-glycosylation modification was detected in the larger band (∼50 kDa) but only contributed to a small part of the extra molecular weight. Other modifications, such as phosphorylation, acetylation and methylation may also occur during exogenous gene expression in maize. The molecular weights of the two bands are higher than their calculated values but lower than that of MAN5A-SST produced in *P. pastoris*. The result indicated that post-translation modification of MAN5AS in maize is much simpler than in yeast. Similar changes have also been reported in other transgenic works [45]. Because no β-mannanase activity was detected in the root, stem and leaf of a positive line, the specific exogenous gene expression in seeds not only increased the value of animal diets, but also lessened the potential impairment to plants.

MAN5AS was biologically active in the range of pH 1.0–7.0 with the peak activity at pH 4.0 and had the highest activity at 90°C (Figure 5a and c). Agrawal *et al.* reported the chloroplast-derived fungal mannanase having the peak activity at pH 5.0 with the optimal temperature of 70°C [27]. Different species of animals have different physiological pHs in stomach and intestine. For example, the pH is 1.3–3.5 in pig stomach and 2.8–4.8 in chicken stomach, and 6.0–7.0 in rumen [46]. Thus an ideal feed β-mannanase should function at pH 1.0–7.0. Thermostability of feed enzyme during the high temperature feed processing is another key criterium. Although MAN5AS and MAN5A-SST are derived from different hosts, both crude enzymes had similar thermostability. Moreover, MAN5AS retained more activities after pelleting (Table 6). Thus MAN5AS represents a favorable candidate for feed enzyme. Similar results that plant-derived enzymes showed better stability have been reported in tobacco [27], [47]. This phenomenon might be ascribed to the different folding patterns and disulphide bond formations in microbes and plants [47].

In summary we successfully constructed a tissue-specific vector for expressing a β-manannase gene in transgenic maize seeds. DNA and protein analysis and enzyme characterization indicated that the β-manannase produced in transgenic maize had high yield, high activity, stable inherence over generations and improved enzyme properties. It is the first time to report the expression of a β-mannanase directly in forage crops on a large scale. Our study provides a new, environment friendly and low-cost approach to produce transgenic maize with social and ecological significance. Once we obtained the security certificate, it will be widely used in feed industry to save cost and energy.

## Supporting Information

Table S1 Composition of the transgenic and non-transgenic maize seeds.
(DOC)
