# Supplementary material for: Overexpression of a Fungal β-Mannanase from Bispora sp. MEY-1 in Maize Seeds and Enzyme Characterization
Source: PLoS One. 2013 Feb 11;8(2):e56146. doi: 10.1371/journal.pone.0056146 (PMC3569411; doi:10.1371/journal.pone.0056146)
Supplement: Table S1 — Composition of the transgenic and non-transgenic maize seeds. (DOC) [file pone.0056146.s001.doc]

**Table S1.** Composition of the transgenic and non-transgenic maize seeds.

|  | **MAN5AS** | **Zheng 58** |  | **MAN5AS** | **Zheng 58** |
| --- | --- | --- | --- | --- | --- |
| Ingredients |  |  | Glycine | 0.31 | 0.34 |
| Moisture | 12.94 | 9.24 | Alanine | 0.65 | 0.72 |
| Protein | 8.68 | 9.70 | Cystine | 0.22 | 0.22 |
| Fat | 2.52 | 3.44 | Valine | 0.40 | 0.45 |
| Fiber | 2.20 | 2.60 | Methionine | 0.23 | 0.24 |
| Ash | 1.30 | 1.40 | Isoleucine | 0.28 | 0.31 |
| Nitrogen free extract | 72.36 | 73.62 | Leucine | 0.95 | 1.11 |
| Amino acids (%) |  |  | Tyrosine | 0.24 | 0.23 |
| Aspartic acid | 0.57 | 0.60 | Phenylalanine | 0.43 | 0.49 |
| Threonine | 0.32 | 0.36 | Histidine | 0.23 | 0.25 |
| Serine% | 0.40 | 0.46 | Lysine% | 0.27 | 0.29 |
| Glutamic acid | 1.37 | 1.55 | Arginine | 0.35 | 0.36 |
| Proline | 0.68 | 0.78 | Tryptophan | 0.07 | 0.07 |
